# Efficacy of early integrated rehabilitation therapy on treatment outcomes and limb functional recovery in patients with cerebral embolism: An observational study

**DOI:** 10.1097/MD.0000000000038436

**Published:** 2024-06-14

**Authors:** Weigu Ban, Peng Qing, Xiuying Teng, Lina Lu, Hui Qi

**Affiliations:** a Rehabilitation Medicine Department, the Second Affiliated Hospital of Heilongjiang University of Chinese Medicine, Harbin, Heilongjiang Province, China; b Department of Acupuncture and Moxibustion, The First Affiliated Hospital of Ji’nan University, Guangzhou, Gungdong Province, China; c Psychological Department, The Second Affiliated Hospital of Heilongjiang University of Traditional Chinese Medicine, Harbin, Heilongjiang Province, China.

**Keywords:** cerebral embolism, early integrated rehabilitation therapy, motor recovery, muscle strength recovery, neurological recovery

## Abstract

Cerebral embolism presents a significant challenge for recovery of motor and neurological function. Early integrated rehabilitation therapy (EIRT) has been proposed as a beneficial approach, yet its efficacy requires thorough evaluation. This retrospective study, conducted from January 2020 to January 2023, involved 117 patient’s post-cerebral embolism, divided into an EIRT group (n = 56) receiving EIRT and a control group (n = 61) receiving standard care. The Fugl-Meyer Assessment (FMA) and the National Institutes of Health Stroke Scale (NIHSS) were used to evaluate motor and neurological functions, while muscle strength was categorized from Level 0 (complete paralysis) to Level V (normal strength) to assess physical recovery. Eligibility centered on confirmed cerebral embolism diagnosis, timing of poststroke admission, and baseline functional status. The study adhered to strict ethical standards, with informed consent obtained from all participants. The EIRT group showed substantial improvements in both FMA and NIHSS scores compared to the control group, indicating better motor and neurological recovery. Significant differences were found in the posttreatment FMA (*P* < .01) and NIHSS scores (*P* < .01). Muscle strength analysis further confirmed the positive impact of EIRT with more patients in the EIRT group achieving higher levels of muscle strength at discharge. The study demonstrates the potential of EIRT to significantly improve motor and neurological outcomes for patient’s post-cerebral embolism. The marked improvements in the observation group suggest that EIRT should be considered for broader application in stroke rehabilitation to enhance recovery and improve quality of life.

## 1. Introduction

Cerebral embolism, a form of ischemic stroke, occurs when a blood clot travels to the cerebral arteries and occludes blood flow to areas of the brain, resulting in significant morbidity and mortality worldwide.^[1]^ The aftermath of this event often leaves individuals with substantial impairments, including motor, cognitive, and psychological deficits.^[2]^ Early integrated rehabilitation therapy (EIRT), encompassing a multidisciplinary approach involving physical, occupational, speech, and sometimes psychological therapies, has emerged as a pivotal component in the post-acute management of cerebral embolism.^[3]^ This approach aims to enhance functional recovery, minimize disability, and improve quality of life.^[4]^ The incidence of cerebral embolism has been on the rise, attributed to an aging population and the prevalence of contributing risk factors such as atrial fibrillation, cardiovascular diseases, and lifestyle factors.^[5]^ Despite advancements in acute medical and surgical interventions, many patients continue to experience significant functional deficits, underscoring the necessity for effective post-acute rehabilitation strategies.^[6]^ Early integrated rehabilitation, initiated soon after acute management, is hypothesized to leverage the brain’s plasticity mechanisms at a time when recovery potential is maximal.^[7]^

EIRT encompasses a range of therapeutic strategies tailored to the individual’s specific impairments and recovery goals.^[8]^ Physical therapy is directed at improving mobility, strength, and coordination, while occupational therapy focuses on restoring the ability to perform daily activities.^[9]^ Speech and language therapy aims to recover communication abilities, and psychological support addresses the emotional and behavioral challenges that may arise poststroke.^[10]^ This integrated approach is designed to be patient-centered, addressing the comprehensive needs of stroke survivors.^[11]^ The rationale for early intervention lies in the concept of neural plasticity – the brain’s ability to reorganize and adapt following injury.^[12]^ In the early stages following a cerebral embolism, the brain is believed to be more receptive to therapeutic interventions, potentially leading to better functional outcomes.^[3]^ However, the timing, intensity, and specific components of EIRT necessary to achieve optimal results remain subjects of ongoing research.

However, the efficacy of early integrated rehabilitation specifically in the context of cerebral embolism remains insufficiently explored. There is a need for comprehensive research to evaluate the effectiveness of these interventions in enhancing functional recovery, particularly in limb function, and in improving overall treatment outcomes. Therefore, this study aims to rigorously assess the impact of EIRT on treatment outcomes and limb functional recovery in patients with cerebral embolism.

## 2. Methods

### 2.1. Study design

An extensive retrospective analysis was conducted at our institution to ascertain the effectiveness of EIRT on treatment outcomes and limb functional recovery in patients with cerebral embolism. The period of this analysis spanned from January 2020 to January 2023. Patients were divided into 2 cohorts: the EIRT group, consisting of 56 individuals who received EIRT, and the control group, comprising 61 individuals who received standard care. Informed consent was obtained from all participants involved in the study. The research methodology, objectives, and protocols underwent thorough review and were approved by the ethics committee of our hospital. This process ensured that the study conformed to the highest ethical standards, maintaining patient confidentiality and rights throughout the investigation.

### 2.2. Inclusion and exclusion criteria

#### 2.2.1. Inclusion criteria

(1) *Diagnosis:* Patients must have a clinically and radiologically confirmed diagnosis of cerebral embolism.
(2) *Time since stroke:* Admission to the study within 3 months poststroke to ensure that rehabilitation therapy is considered early.
(3) *Functional status:* Eligible participants must have a definable level of motor and neurological function, quantitatively assessed using the Fugl-Meyer Assessment (FMA) and the National Institutes of Health Stroke Scale (NIHSS), respectively, with specific ranges set for inclusion and exclusion based on these assessments.
(4) *Consent:* Patients must be able and willing to provide informed consent to participate in the study and undergo rehabilitation therapy.

#### 2.2.2. Exclusion criteria

(1) *Previous neurological disorders:* Patients with a history of neurological impairments or conditions that may influence rehabilitation outcomes, such as previous strokes or severe neurodegenerative diseases.
(2) *Medical instability:* Patients with medical conditions that are not stable enough to participate in rehabilitation therapy, including severe cardiovascular, renal, or hepatic diseases.
(3) *Concurrent interventions:* Patients currently receiving other experimental treatments or interventions that might interfere with the evaluation of EIRT.
(4) *Cognitive impairment:* Severe cognitive impairments or psychiatric conditions that would preclude understanding instructions or informed consent.
(5) *Life expectancy:* Patients with a life expectancy of <1 year due to other medical conditions, as this could confound the long-term assessment of rehabilitation outcomes.

### 2.3. Study groups and treatment protocols

Patients admitted with cerebral embolism were divided into 2 groups: a control group and an observation group, with both receiving standard symptomatic treatments including blood pressure adjustment, respiratory support, brain edema symptom management, infection prevention, venous thrombosis prophylaxis in the lower limbs, and nutritional support.^[13]^ Additionally, once their condition stabilized, the control group received conventional rehabilitation, which encompassed physical therapy to enhance limb mobility, speech therapy, and swallowing rehabilitation to aid in their recovery process.

In the observation group, patients underwent EIRT, which encompassed a multifaceted approach tailored to their specific needs. The therapy initiation involved psychological counseling sessions aimed at gaining insights into their condition, addressing concerns, and fostering a positive mindset toward recovery, with active involvement of family members for emotional reinforcement. Subsequently, a comprehensive assessment was conducted to evaluate the patient’s physical health, psychological well-being, family dynamics, financial circumstances, and social support network. This assessment included detailed evaluations of balance, activities of daily living (ADL), manual muscle strength, and residual motor function to formulate a personalized and comprehensive EIRT plan.

The EIRT protocol began 48 hours after initial treatment and included a variety of therapeutic interventions. Limb rehabilitation involved a gradual increase in joint movements, starting with passive motions and advancing to more active exercises, focusing on improving movement amplitude, strength, and duration. Speech therapy included exercises like cheek puffing, tongue clicking, and lip pursing, followed by simple phonation tasks, encouraging patients to use familiar language and express their thoughts freely. Swallowing training was enhanced for patients with dysphagia, promoting self-feeding and proper swallowing techniques. Lastly, postural and locomotion training commenced with sitting and posture control, progressively moving to standing and walking exercises as muscle strength improved, utilizing support aids like balance bars or canes to assist in maintaining stability. Each of these components was carefully integrated into the rehabilitation process to ensure a comprehensive approach to the patient’s recovery.

### 2.4. Assessment of motor and neurological recovery poststroke

Observational metrics for the study utilized the FMA and the NIHSS to quantitatively assess motor and neurological functions pre-rehabilitation and post-rehabilitation. The FMA, a stroke-specific index, evaluates motor functioning, balance, sensation, and joint function in stroke patients, emphasizing physical impairment and recovery. It is detailed into sections for movement, reflexes, and coordination, culminating in a score depicting motor capabilities. The NIHSS is a comprehensive tool measuring neurological deficits common in stroke, including consciousness, motor strength, gaze, and language abilities, among others. It is crucial for establishing baselines and tracking recovery. Muscle strength was categorized from Level 0, complete paralysis, to Level V, normal strength, indicating varying degrees of recovery.

### 2.5. Statistical analysis

Statistical evaluations were conducted using SPSS software, version 27.0, with a structured approach to data categorization and analysis. Initially, all data underwent normality testing and were classified as either quantitative or categorical. Quantitative data adhering to normal distribution were analyzed for differences between groups using independent sample *t* tests, with results presented as mean ± standard deviation (mean ± SD). For comparisons across multiple groups, 1-way ANOVA was employed, followed by post hoc analysis using the LSD *t* test. Welch correction was utilized for instances of unequal variances. Categorical data, represented as frequencies and percentages, were examined using the chi-square (χ^2^) test to determine relationships or independence among categorical variables. The study adopted a 2-tailed hypothesis testing approach, considering a *P* value of <.05 as indicative of statistical significance.

## 3. Results

### 3.1. Patient demographics and clinical profiles

In this study, patients were allocated into 2 primary groups based on the treatment received. The observation group, consisting of 56 patients, underwent EIRT. This group comprised 30 males and 26 females, with an age range of 50 to 79 years and a mean age of 62.6 ± 3.8 years. The duration of their illness varied from 1 to 6 years, averaging at approximately 2.65 years. The control group included 61 individuals who received standard treatment protocols. The gender distribution in this group was 32 males to 29 females, with ages ranging from 51 to 80 years. The mean age for this group was 62.6 ± 3.6 years, and the duration of illness ranged from 1 to 6 years, averaging at approximately 2.85 years (Table 1). These demographic distributions are critical in ensuring the comparability of the 2 groups and understanding the impact of EIRT compared to conventional treatment methods.

**Table 1:** Demographic and clinical characteristics of the study participants.

Group	Total patients	Gender ratio (M:F)	Age range	Mean age (yrs)	Disease duration range (yrs)	Mean disease duration (yrs)
EIRT	56	30:26	50 to 79	62.6 ± 3.8	1 to 6	2.65
Control	61	32:29	51 to 80	62.6 ± 3.6	1 to 6	2.85

EIRT = early integrated rehabilitation therapy, F = female, M = male.

### 3.2. Comparative analysis of rehabilitation outcomes

In this study, we assessed the efficacy of rehabilitation interventions in patients with cerebral embolism using 2 established metrics: the FMA for motor recovery and the NIHSS for neurological deficit. Patients were divided into an observation group, receiving EIRT, and a control group, receiving standard care. The observation group demonstrated a significant improvement in motor function, as evidenced by an increase in the FMA scores from 66.3 ± 7.5 pretreatment to 90.2 ± 7.5 posttreatment. Neurological status also improved markedly, with NIHSS scores decreasing from 24.9 ± 2.8 to 14.2 ± 1.5. In contrast, the control group showed modest improvements; their FMA scores rose from 63.9 ± 8.6 to 71.1 ± 6.5, and NIHSS scores decreased from 26.1 ± 2.6 to 20.3 ± 2.1. The statistical analysis indicated a significant difference in posttreatment scores between the groups (*P* < .01 for both FMA and NIHSS), affirming the superior efficacy of the integrated rehabilitation approach over standard care (Table 2).

**Table 2:** Comparison of pretreatment and posttreatment FMA and NIHSS scores between patient groups.

Group	FMA pretreatment (Mean ± SD)	FMA posttreatment (Mean ± SD)	NIHSS pretreatment (Mean ± SD)	NIHSS posttreatment (Mean ± SD)
EIRT	66.3 ± 7.5	90.2 ± 7.5	24.9 ± 2.8	14.2 ± 1.5
Control	63.9 ± 8.6	71.1 ± 6.5	26.1 ± 2.6	20.3 ± 2.1
*t* Value	0.98	2.76	0.96	2.67
*P* value	.38	<.01	.85	<.01

EIRT = early integrated rehabilitation therapy, FMA = Fugl-Meyer assessment, NIHSS = National Institutes of Health Stroke Scale.

### 3.3. Comparative analysis of muscle strength at discharge

In this segment of the study, muscle strength outcomes for stroke patients undergoing rehabilitation were quantified and categorized across 6 defined levels (Level 0 to Level V), representing a spectrum from complete paralysis to normal muscle strength. The assessment involved 2 distinct groups: an observation group, receiving a specific rehabilitation treatment, and a control group, provided with standard care. The observation group showed favorable recovery patterns, with no individuals remaining at Level 0 (complete paralysis) posttreatment. A significant proportion of patients in this group achieved substantial recovery, as indicated by 44.6% reaching Level V (normal muscle strength). Intermediate recovery levels (Levels I–IV) were distributed amongst the remaining participants, suggesting varied individual responses to the treatment. In contrast, the control group exhibited a different recovery trajectory. Notably, 11.5% remained at Level 0, and the majority (57.4%) were categorized at Level I, indicating a predominance of minimal recovery. A smaller fraction of patients reached full recovery (Level V), underscoring a generally less effective outcome compared to the observation group (Table 3).

**Table 3:** Comparative analysis of muscle strength levels in patients at discharge.

Group	Level 0 (n; %)	Level I (n; %)	Level II (n; %)	Level III (n; %)	Level IV (n; %)	Level V (n; %)
EIRT	0 (0%)	5 (8.9%)	12 (21.4%)	3 (5.4%)	11 (19.6%)	25 (44.6%)
Control	7 (11.5%)	35 (57.4%)	2 (3.3%)	3 (4.9%)	5 (8.2%)	9 (14.8%)

EIRT = early integrated rehabilitation therapy.

## 4. Discussion

The pivotal role of rehabilitation in the aftermath of a cerebral embolism cannot be overstated.^[14]^ The quest to improve treatment outcomes and limb functional recovery for patients has led to the exploration of EIRT.^[15]^ This approach is grounded in the understanding that the period following a cerebral embolism is critical; the brain’s plasticity is heightened, and timely, comprehensive intervention can significantly influence the trajectory of recovery.^[16]^ EIRT represents a paradigm shift from traditional, later-stage rehabilitation efforts, emphasizing the importance of initiating therapy as soon as clinically feasible post-event.^[17]^ The efficacy of EIRT is derived from its holistic nature, addressing not only the physical impairments typically associated with cerebral embolism but also the cognitive and emotional aspects that significantly impact recovery.^[18]^ By integrating various therapeutic modalities – physical therapy, occupational therapy, speech and language therapy, and psychological support – EIRT aims to provide a comprehensive, patient-centered care plan.^[19]^ This integrated approach ensures that the multifaceted needs of each patient are met, promoting better overall outcomes and enhancing quality of life. As healthcare professionals and researchers continue to seek optimal strategies for poststroke recovery, understanding and harnessing the potential of EIRT becomes increasingly important.^[20]^ The discussion around its efficacy is not just academic; it is about redefining recovery possibilities and ensuring that every patient has access to the best possible care at the most opportune time.

This study’s results bring forth compelling evidence supporting the effectiveness of EIRT in enhancing both motor and neurological recovery post-cerebral embolism. By comparing patients’ outcomes in 2 distinct cohorts – those receiving EIRT and those subjected to standard care – significant differences in recovery trajectories were elucidated. The observation group demonstrated notable improvements in FMA scores from 66.3 ± 7.5 pretreatment to 90.2 ± 7.5 posttreatment, and NIHSS scores from 24.9 ± 2.8 to 14.2 ± 1.5. Conversely, the control group showed more modest improvements. These findings provide a quantitative testament to the potential benefits of adopting an early, integrated approach to rehabilitation following cerebral embolism.

The study’s methodology, focusing on early intervention, taps into the critical period following cerebral insult when the brain’s plasticity mechanisms are most receptive to change. Neuroplasticity, the underlying principle of recovery, allows for functional reorganization and adaptation in response to rehabilitation efforts. EIRT, with its multidisciplinary focus, provides a comprehensive stimulus for such plastic changes, encompassing not only physical rehabilitation but also cognitive and emotional support. The tailored, patient-centric nature of EIRT ensures that each individual’s unique recovery needs and potential are addressed, leading to more effective and sustainable outcomes. Moreover, the observed improvements in muscle strength, particularly the high percentage of EIRT group patients achieving Level V recovery, indicate a robust response to the intervention. The contrast with the control group’s predominance at lower recovery levels (with 11.5% remaining at Level 0 and 57.4% at Level I) starkly highlights the relative efficacy of the integrated approach over standard care.

The implications of these findings for clinical practice are multifold. First, they underscore the need for early assessment and intervention poststroke to maximize recovery potentials. Clinicians should be aware of the time-sensitive nature of neuroplasticity and strive to initiate rehabilitation as soon as possible. Second, the demonstrated superiority of EIRT suggests that a holistic, integrated approach to poststroke care should be the norm rather than the exception. Rehabilitation programs should encompass a range of therapeutic modalities, addressing not just physical but also cognitive and emotional aspects of recovery. Personalization of care, adapting strategies to fit individual patient profiles and progress, should be a standard practice. Furthermore, the study’s findings advocate for more extensive patient and family education and involvement in the rehabilitation process. Psychological counseling and family support were integral components of EIRT, contributing to improved motivation, engagement, and ultimately, better outcomes. Encouraging active participation and emotional support from family members can significantly enhance the rehabilitation experience and its effectiveness.

This study has several limitations that warrant consideration. First, the retrospective nature of the analysis may introduce biases related to data collection and patient selection. Future studies could benefit from a prospective, randomized controlled design to validate the findings and minimize biases. Second, the study’s limited follow-up period provides a snapshot of the recovery process but does not capture long-term outcomes. Extending the follow-up duration would allow for assessment of the sustained impacts of EIRT and its effect on long-term quality of life. Third, our study did not include a risk-benefit analysis using logistic regression to quantitatively evaluate the magnitude of benefits through odds ratios and confidence intervals. Incorporating this analytical approach in future studies could provide a clearer understanding of the intervention’s impact and address potential confounders more robustly. Additionally, exploring the individual components of EIRT to determine their specific effectiveness and contributions to therapy success, and expanding the study to include a more diverse patient population, would enhance the generalizability of the findings. Understanding patient-specific factors, such as age, stroke severity, and pre-stroke functional status, can help tailor EIRT more effectively to individual needs, leading to optimized treatment strategies and better patient outcomes.

## 5. Conclusions

This study demonstrates that EIRT significantly enhances the recovery of impaired neurological functions, improves motor skills, and increases muscle strength in patients with acute cerebral embolic stroke. The evident efficacy of this approach in early stages poststroke highlights its potential in transforming patient outcomes. Therefore, it merits consideration for broader application and integration into standard poststroke care practices to optimize recovery and improve the quality of life for stroke survivors.

## Author contributions

**Conceptualization:** Weigu Ban, Hui Qi.

**Formal analysis:** Weigu Ban, Lina Lu.

**Investigation:** Weigu Ban, Lina Lu.

**Writing – original draft:** Weigu Ban.

**Data curation:** Peng Qing.

**Funding acquisition:** Peng Qing.

**Methodology:** Peng Qing, Xiuying Teng.

**Resources:** Xiuying Teng.

**Software:** Xiuying Teng.

**Supervision:** Hui Qi.

**Writing – review & editing:** Hui Qi.
